# Von Economo neurons are part of a larger neuronal population that are selectively vulnerable in C9orf72 frontotemporal dementia

**DOI:** 10.1111/nan.12558

**Published:** 2019-06-10

**Authors:** P. Gami‐Patel, I. van Dijken, J. C. van Swieten, Y. A. L. Pijnenburg, Netherlands Brain Bank, A. J. M. Rozemuller, J. J. M. Hoozemans, A. A. Dijkstra

**Affiliations:** ^1^ Department of Pathology Amsterdam Neuroscience Amsterdam University Medical Centre Location VUMC Amsterdam The Netherlands; ^2^ Department of Neurology Alzheimer Centre Erasmus MC Rotterdam The Netherlands; ^3^ Department of Neurology Alzheimer Centre Amsterdam Neuroscience Amsterdam University Medical Centre Location VUMC Amsterdam The Netherlands; ^4^ Netherlands Institute for Neuroscience Amsterdam The Netherlands

**Keywords:** *C9orf72*, frontotemporal dementia, GABRQ, social‐emotional behaviour, von Economo neuron

## Abstract

**Aims:**

The behavioural variant of frontotemporal dementia with a *C9orf72* expansion (C9‐bvFTD) is characterised by early changes in social‐emotional cognition that are linked to the loss of von Economo neurons (VENs). Together with a subset of neighbouring pyramidal neurons, VENs express the GABA receptor subunit theta (GABRQ). It is not known if the selective vulnerability of VENs in C9‐bvFTD also includes this GABRQ‐expressing population.

**Methods:**

We quantified VENs and GABRQ immunopositive neurons in the anterior cingulate cortex (ACC) in C9‐bvFTD (*n* = 16), controls (*n* = 12) and Alzheimer's disease (AD) (*n* = 7). Second, we assessed VENs and GABRQ‐expressing populations in relation to the clinicopathological profiles.

**Results:**

We found the number of VENs and GABRQ‐expressing neurons and their ratio over the total layer 5 neuronal population was lower in C9‐bvFTD compared to control and AD. C9‐bvFTD donors with underlying TDP43 type A pathology in the ACC showed the highest loss of GABRQ‐expressing neurons. C9‐bvFTD donors that did not present with motor neuron disease (MND) symptoms in the first half of their disease course showed a prominent loss of GABRQ‐expressing neurons compared to controls. C9‐bvFTD donors with no symptoms of psychosis showed a higher loss compared to controls. Across all donors, the number of VENs correlated strongly with the number of GABRQ‐expressing neurons.

**Conclusion:**

We show that VENs, together with GABRQ‐expressing neurons, are selectively vulnerable in C9‐bvFTD but are both spared in AD. This suggests they are related and that this GABRQ‐expressing population of VENs and pyramidal neurons, is a key modulator of social‐emotional functioning.

## Introduction

The behavioural variant of frontotemporal dementia (bvFTD) is the main clinical manifestation of frontotemporal lobar degeneration (FTLD) 1. In up to 20% of cases the disease is familial, with a repeat expansion in the *C9orf72* gene being the most frequent genetic cause of bvFTD (C9‐bvFTD) 2. Patients with the expansion can present with a spectrum of symptoms, including motor neuron disease (MND)/amyotrophic lateral sclerosis (ALS) 2. Psychotic symptoms, such as delusions and hallucinations, are also commonly seen in C9‐bvFTD patients, during or prior to the onset of dementia 3. Pathologically, in patients with C9‐bvFTD the aggregation of phosphorylated transactive response DNA binding protein 43 kDa (TDP43) is most likely linked to neurodegeneration 4. This TDP43 pathology has been subdivided into distinct subclasses, types A‐E, based on its cortical laminar distribution and morphology 5. In C9‐bvFTD, the commonly observed subtypes are TDP43 type A and B 6, 7. The clinical relevance of the different laminar distribution patterns within the C9‐bvFTD donors is currently unclear.

Clinically, progressive social‐emotional processing deficits are one of the earliest symptoms seen in bvFTD, and have been linked to a specific neuronal type, the von Economo neurons (VENs) 8, 9. VENs are selectively vulnerable in the earliest stages of the disease and have been implicated in the behavioural symptoms of bvFTD 8, 9, 10, 11, 12. VENs have a unique morphology and are found mainly in layer 5 of the human anterior cingulate cortex (ACC) and frontoinsular cortex (FI) 13. They are distinguished from pyramidal neurons by their large bipolar cell body and thick dendrites 14. VENs have been identified with a similar regional distribution in highly social mammals, such as primates, cetaceans and elephants, but are not found in common laboratory animals, such as mice and rats 15, 16, 17. Together with a subset of neighbouring layer 5b pyramidal neurons in the ACC and FI, VENs possess a monoaminergic phenotype and express the GABA receptor subunit theta (GABRQ) and are therefore part of a unique cortical neuronal population 18. Currently, it is not known if the selective vulnerability of VENs in bvFTD includes the neighbouring GABRQ‐expressing pyramidal neurons.

Here, we aim to investigate if VENs are part of a larger neuronal population by quantifying the selective vulnerability of VENs and related GABRQ‐expressing neurons in C9‐bvFTD in the ACC. In addition, we aim to study the clinicopathological relationships with a focus on the GABRQ‐expressing population and VENs.

## Materials and methods

### Subjects

Post‐mortem brain tissue was obtained from the Netherlands Brain Bank and the department of pathology, Amsterdam University Medical Centre, location VUmc, Amsterdam, The Netherlands. Donors were seen at either VUmc or Erasmus Medical Centre, Rotterdam, The Netherlands. We included patients with bvFTD as a result of *C9orf72* mutation (genetically confirmed at Erasmus Medical Centre, Rotterdam, *n* = 16), donors with typical amnestic presentation of Alzheimer's disease (AD) (*n* = 7), and age‐matched neurologically unaffected controls (*n* = 12) (Table 1 and Table S1). Donors with concomitant pathology were excluded from the study. Extensive clinical reports were available from all donors.

**Table 1:** Demographic and clinical data of donors

	*n*	M/F	Age	Disease duration
C9‐bvFTD	16	6/10	66.5 (40–77)	7 (2–11)
AD	7	4/3	82 (69–96)	8 (3–12)
Control	12	6/6	64 (51–86)	n/a

### Sample selection

Within the ACC, VENs have a greater decrease in a rostrocaudal gradient 19. Therefore, we sampled the ACC in a consistent manner where we took the ACC adjacent to the genu to minimize variation. After 4 weeks of fixation in formalin, the material was embedded in paraffin and cut into a series of sequential 10 μm sections.

### Immunohistochemical procedures

On the sequential slides, immunohistochemical procedures were performed with antibodies against GABA receptor subunit epsilon (GABRE: HPA045918, Sigma Aldrich, St. Louis, MO) that was used for outlining layer 5a, GABRQ (HPA002063; Sigma Aldrich) and pTDP43 (CAC‐TIP‐PTD‐M01, Cosmo Bio, Japan). Briefly, slides were deparaffinised and then incubated in 0.3% H_2_O_2_ in phosphate buffer saline (PBS; pH 7.4) for 30 min to block endogenous peroxidase activity. Sections were then washed with PBS (3 × 5 min) and heat‐induced antigen retrieval was performed in citrate buffer (pH 6.0) using the autoclave (121°C for 5 min). After washing, sections were incubated in 10% normal goat serum in PBS (1:50) for 30 min followed by incubation in primary antibody (GABRQ 1:750; GABRE 1:1000, pTDP43 1:8000) for 1 h (pTDP43 and GABRE) or overnight at 4°C (GABRQ). After washing, sections were incubated with HRP‐labelled Envision (K5007; DAKO, Glostrup, Denmark), washed and then visualization of immunostaining was seen with chromogen 3,3′‐diaminobenzidine (DAB; K5007; DAKO). Finally, sections were counterstained with haematoxylin, dehydrated, and cover slipped with Quick‐D (Klinipath, Duiven, The Netherlands). Negative controls for each primary antibody were included by omitting the primary antibody and showed no immunoreactivity, brainstem sections were used for positive controls for GABRE and GABRQ 18.

### Quantification of GABRQ‐expressing cortical neurons

**Figure 1:**
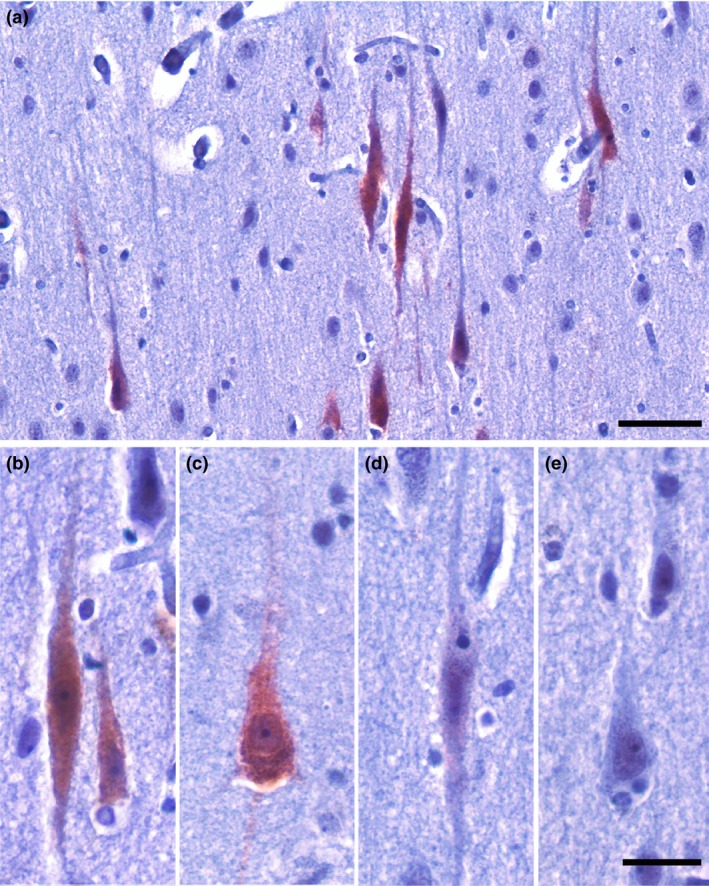
GABRQ expression in the ACC. GABRQ expression in layer 5b of the ACC (**a**). GABRQ is expressed in VENs (**b**) and a subset of neighbouring pyramidal neurons (**c**) but is not seen in all layer 5b pyramidal neurons (**e**). Though infrequent, GABRQ‐negative VENs are also present (**d**) (14). Scale bars represent 100 μm (**a**) and 25 μm (**b–e**).

To quantify GABRQ expression, we analysed the GABRQ stained slides from the ACC using Stereoinvestigator software (V11.6.2). First, the cortex was outlined using the grey‐white matter border. Second, layer 5 was delineated using one slide stained with GABRE and the sequential section with GABRQ, where GABRE depicted the apical layer 5 border and GABRQ the lower layer 5b border (Figure S1). Within layer 5, all neurons were counted using the Meander scan option in Stereoinvestigator and divided into 4 categories: (1) GABRQ‐expressing VENs, (2) GABRQ‐expressing pyramidal neurons, (3) GABRQ‐negative VENs and (4) GABRQ‐negative pyramidal neurons (Figure 1). VENs were identified based on their typical morphological profile 19: a long elongated soma with one apical and one basal dendrite. In contrast, pyramidal neurons were identified based on their more rounded or tear drop soma with two basal dendrites and were counted if their cell body was larger than 10 μm.

### Quantification of TDP43 pathology

A sequential slide was used to identify the molecular subclass of TDP43 pathology and quantify the burden of phosphorylated TDP43 (pTDP43) pathology in layer 5. Briefly, donors were classed as type A when inclusions and short threads were observed to be predominantly in the superficial layers. If mainly cytoplasmic inclusions were seen with no preference for layers, type B was denoted. If long threads were seen predominantly in the superficial layers type C was characterised. Type D was given if frequent lentiform intranuclear inclusions were seen. Finally, if the ACC showed a strong granular intracellular and extracellular spread with no intracellular inclusions, type E was considered 5. The burden of pathology in layer 5 was quantified using the Meander scan option in Stereoinvestigator. The outline of the delineation was adjusted to the slightly different plane of tissue. The types of pathology were scored separately: threads larger than 10 μm, extracellular aggregates, intraneuronal large round aggregates and intraneuronal punctate granular aggregates 5. The burden was defined as the combined scores of all types of pTDP43 pathology.

### Clinical features

The medical history was used to determine the medical profile of the donors. For controls, no neurological symptoms were recorded. For C9‐bvFTD, the following symptoms were noted as present if donors presented with them in the first half of their disease course: memory symptoms, speech symptoms, and signs of MND 20, 21. Symptoms of psychosis, such as delusions and hallucinations, were considered present if they were reported at any given time during the disease progression. The presence of the symptoms was used to subdivide the C9‐bvFTD donors based on their clinical profile.

### Statistics

Statistics were performed using SPSS 22. Pearson correlation was used to assess correlation and differences between groups were assessed using one‐way ANOVA with Tukey's post‐hoc analysis.

## Results

### Numbers of VENs and GABRQ‐expressing neurons are correlated and selectively vulnerable in C9‐bvFTD

**Figure 2:**
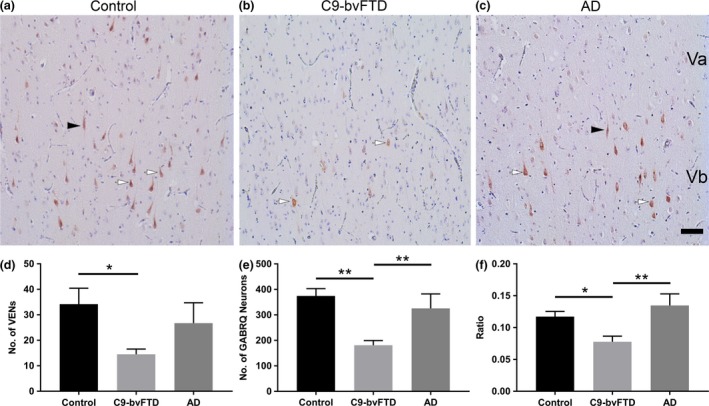
VENs and GABRQ‐expressing neurons are reduced in C9‐bvFTD. The GABRQ‐expressing population is reduced in C9‐bvFTD compared to controls and AD, the black arrowheads indicate GABRQ‐positive VENs and the white arrows GABRQ‐positive pyramidal neurons (**a–c**). Quantification of the number of VENs showed a significant reduction in C9‐bvFTD compared to control (**d**). Similarly, in C9‐bvFTD there was a significant reduction in number of GABRQ‐expressing neurons (VENs and pyramidal) compared to control and AD (**e**). The ratio of GABRQ‐expressing neurons over the total number of neurons in layer 5 showed GABRQ‐expressing neurons were selectively targeted in C9‐bvFTD, with a significant reduction seen when compared to controls and AD (**f**). Scale bar represent 50 μm. Error bars represent standard error of the mean (SEM). *P < 0.05, **P < 0.01.

In this study we quantified the number of VENs and GABRQ‐expressing neurons (VENs and pyramidal neurons) in the ACC of 16 C9‐bvFTD, 12 control and 7 AD donors. We found that on average there was a 57% reduction in number of VENs in C9‐bvFTD when compared to control (*P *= 0.01) (Figure 2 **d**). Using an ANOVA, a significant difference was seen in the number of VENs present between the three groups; controls, AD and C9‐bvFTD (*F*(2) = 4.92, *P *= 0.01). A similar finding was also seen in the number of GABRQ‐expressing neurons, with a 51% reduction seen in C9‐bvFTD compared to control, which was statistically significant (*P* < 0.001) (Figure 2 **e**). No significant difference in GABRQ‐expressing neurons between AD and controls was detected. This indicates that the GABRQ‐expressing population is spared from neurodegeneration in AD and selectively vulnerable in C9‐bvFTD. No significant difference in total neuronal population of layer 5 was found (*F*(2) = 2.20, *P *= 0.13).

To determine if VENs are part of a larger GABRQ‐expressing population we correlated the total number of VENs with the total number of GABRQ‐expressing neurons and found there was a significant correlation seen across all donors included in this study (*P *= 0.65, *P* < 0.001).

The ratio of GABRQ‐expressing neurons was calculated by dividing the total number of GABRQ‐expressing neurons over the total layer 5 neuronal population. Using an ANOVA, a significant difference was found in ratio between the three groups (*F*(2) = 7.72, *P *= 0.02). Tukey post‐hoc analysis revealed a significant decline of ratio in donors with C9‐bvFTD compared to controls (*P *= 0.02) and compared to AD donors (*P *= 0.004) (Figure 2 **f**).

### Relation between VENs and GABRQ‐expressing neurons and TDP43 pathology

**Figure 3:**
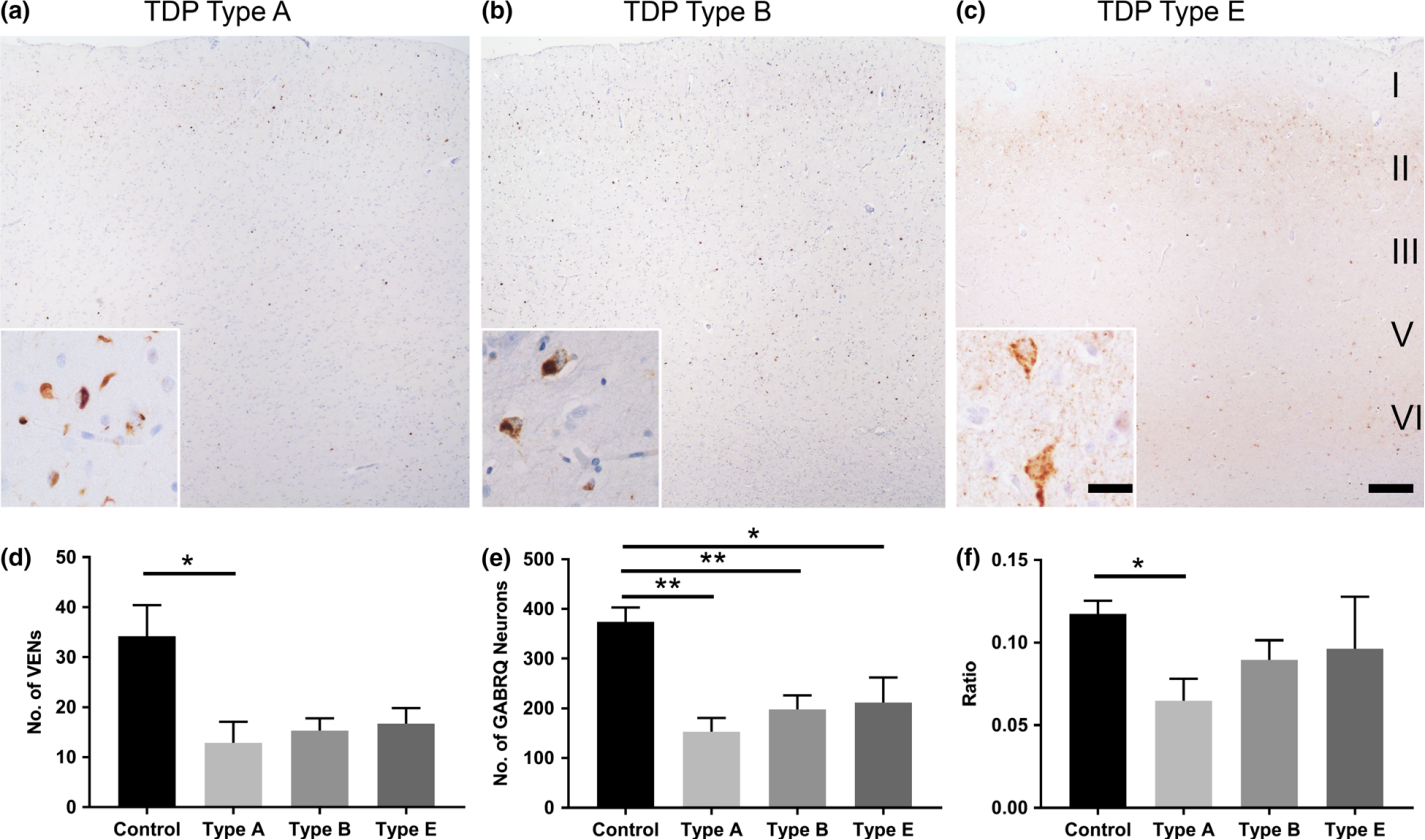
TDP43 type A pathology is related to a higher loss of VENs and GABRQ‐expressing neurons. In the ACC, TDP43 subtypes were characterised and classified accordingly, where TDP43 type A (**a**) was given when cytoplasmic inclusions and threads (**a** inset) were observed predominantly in layer 2. TDP43 type B distribution (**b**) was given if mainly cytoplasmic inclusions (**b** inset) with no preference for a layer was seen. TDP43 type E (**c**) was considered if a strong granular intracellular and extracellular staining (**c** inset) was seen with no intracellular inclusions. A similar reduction in number of VENs was seen across all TDP43 types, with type A reaching significance (**d**). The number of GABRQ‐expressing neurons followed the same trend as VENs, with all reaching significance (**e**). Donors with TDP43 type A pathology had the greatest decline in ratio when compared to control (**f**). Though a reduction is also seen in cases with TDP43 type B and type E this was less prominent and did not reach significance. Scale bars represent 50 μm (**a–c**) and 25 μm (insets). Error bars represent SEM. *P < 0.05, **P < 0.01.

Since TDP43 is believed to play an integral role in neurodegeneration, we next looked at the TDP43 pathology in the ACC 5. Of the 16 donors with C9‐bvFTD TDP43 pathology, seven were subdivided into molecular subclass TDP43 type A, six TDP43 type B, and three TDP43 type E. When we split the groups according to their molecular subclass we found there was a reduction in number of VENs across all subclasses with type A showing the greatest loss (*P *= 0.046) (Figure 3 **d**). A comparable pattern was also seen in the number of GABRQ‐expressing neurons, with all reaching significance (type A: *P* < 0.001; type B: *P *= 0.002; type E: *P *= 0.04) (Figure 3 **e**). In contrast, TDP43 type A donors revealed the largest drop in the ratio of GABRQ‐expressing neurons compared to controls whereas TDP43 type B and type E showed a less prominent loss (*P *= 0.01) (Figure 3 **f**). Using the layer 5 outline from the GABRQ‐quantification, pTDP43 pathology was quantified on a sequential slide. The different types and burden of pathology did not correlate with the ratio and neuronal loss. pTDP43 inclusions in VENs were only seen in 1 of the 16 cases analysed, indicating that in our cohort not the burden, but the distribution as defined by molecular subclasses is related to the GABRQ‐expressing neuronal loss.

### Relation between VENs and GABRQ‐expressing neurons and clinical features

**Figure 4:**
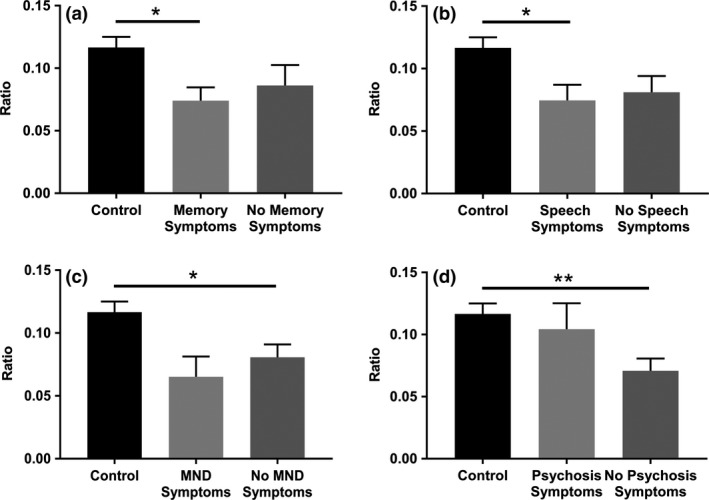
Decreased ratio of GABRQ‐expressing neurons is associated with clinical features. C9‐bvFTD donors presenting with memory symptoms (**a**) and speech symptoms (**b**) but not MND symptoms (**c**) in the first half of their disease course had a significantly lower ratio of GABRQ‐expressing neurons compared to controls. Donors without psychotic symptoms had a reduction in the ratio of GABRQ‐expressing neurons compared to controls, which was not seen in donors with psychotic symptoms (**d**). Error bars indicate SEM. *P < 0.05, **P < 0.01.

In our C9‐bvFTD cohort, disease duration varied from 2 to 11 years (median 7). We investigated the presence of non‐behavioural symptoms in the first half of the donors’ disease course. A lower ratio of GABRQ‐expressing neurons was observed in C9‐bvFTD donors without MND symptoms present in the first half of their disease course compared to controls (*P *= 0.03). This difference was not seen in C9‐bvFTD donors with these symptoms present in the first half of their disease course (*P *= 0.05). In contrast, C9‐bvFTD donors presenting with memory and speech symptoms in the first half of their disease course had a lower ratio of GABRQ‐expressing neurons compared to control (*P *= 0.01 and *P *= 0.02) (Figure 4 **a–c**). Four donors with C9‐bvFTD reported psychotic symptoms throughout their disease duration. The ratio of GABRQ‐expressing neurons was significantly lower in donors with no psychosis present throughout their disease duration compared to controls (*P* < 0.01), which was not observed in donors with presence of psychotic symptoms (*P *= 0.80; Figure 4 **d**) 10.

## Discussion

Here, we have shown that the selective vulnerability of VENs in C9‐bvFTD extends to the larger population of cortical GABRQ‐expressing neurons in the ACC. The selective vulnerability of VENs in bvFTD and other diseases with altered social cognition has been widely discussed in literature 8, 9, 10, 11, 12, 22, 23, 24 but this is the first work to show that VENs are part of a larger population of neurons linked to social cognition.

### VENs and GABRQ‐expressing neurons are part of a similar neuronal population

We quantified the number of VENs and GABRQ‐expressing neurons (VENs and pyramidal neurons) in all donors and found that the selective vulnerability of VENs in C9‐bvFTD is shared with GABRQ‐expressing neurons and that this GABRQ‐expressing neuronal population is spared in AD, similar to what has been reported for VENs 8, 9, 10, 11. Moreover, a significant correlation is seen between VEN number and GABRQ‐expressing neurons across healthy donors and donors with AD and C9‐bvFTD. This indicates that the loss and sparing of these neuronal types in dementia occurs concurrently, and supports the hypothesis that VENs and GABRQ‐expressing neurons are related and together form a larger cortical population 18. Additionally, the sparse expression of GABRQ in pyramidal neurons in layer 5 in the frontal and temporal cortex is in line with the recent uncovering of sparse VENs seen in the frontal cortex 18, 25, 26. It is possible that the GABRQ‐expressing neuronal population also includes a small number of neurons outside the ACC and FI.

Due to their selective vulnerability, regional distribution and phylogenetic distribution among different species, VENs have been linked to higher social cognition 8, 9, 10, 15, 17, 19, 27, 28, 29. In other diseases where social cognition is altered such as autism and schizophrenia, the numbers of VENs are also altered compared to control donors 22, 23, 24, 30. Our findings suggest that the neuronal population involved in these complex social processes is larger than initially assumed. This provides opportunities to expand the study of VENs and GABRQ‐expressing neurons in greater detail across diseases where social cognition is altered.

The biochemical similarities of VENs and GABRQ‐expressing neurons also include the expression of vesicular monoamine transporter 2 (VMAT2) 18. The monoaminergic properties and a specialized GABA receptor subtype reveal that these neurons could relay information in a unique manner distinct to their neighbouring neurons. This study utilized immunohistochemistry to visualize GABRQ expression and is therefore limited as low levels of protein expression cannot be visualized. A small number of VENs do not show detectable expression levels of GABRQ using immunohistochemistry, however, it is unclear if these neurons do not express GABRQ at all. Although unlikely, one possibility could be that the neighbouring GABRQ‐negative neurons in the C9‐bvFTD donors have simply lost their expression or do not reach detectable levels of GABRQ as a result of disease pathogenesis, and therefore cannot be distinguished from other layer 5b pyramidal neurons that never expressed the subunit. Loss of receptor expression would result in an uncoupling of signalling pathways and functionality of the neurons. We therefore hypothesize that the effect of neurodegeneration or loss of GABRQ expression would have similar effects on the signal processing.

### GABRQ‐expressing neurons are selectively vulnerable in donors with C9‐bvFTD with different pathological subclasses

In our analysis, we have shown that donors with underlying TDP43 type A pathology in the ACC have a higher loss of GABRQ‐expressing neurons in layer 5 compared to TDP43 types B and E, with TDP43 type E donors having the least GABRQ‐expressing neuronal loss. This indicates that within the clinical spectrum, there is also a pathological spectrum where patients with distinct local TDP43 aggregation distribution patterns also display different patterns of neuronal loss. It has been suggested that the TDP43 type E pathology represents a MND‐related state of TDP43 pathology 31, 32, however in our cohort, none of the donors with granular pathology in the ACC displayed prominent MND symptoms. No contribution of layer 5 pTDP43 burden on neuronal loss was found in the ACC and remarkably, only one case showed neuronal inclusions in VENs. A recent study reported that though TDP43 inclusions were much lower in C9‐bvFTD/MND compared to sporadic bvFTD, VENs were fourfold more prone to TDP43 inclusions compared to their neighbouring layer 5 neurons in C9‐bvFTD/MND in the FI 33. In these C9‐bvFTD/MND donors they also reported that VENs lacking nuclear TDP43 and TDP43 inclusions, termed TDP43‐depleted neurons, were just as frequent as those with inclusions, however this varied between donors 33. Based on these findings, it is possible that our C9‐bvFTD donors possess TDP43‐depleted neurons, which could explain why we did not observe inclusions in VENs. It is also important to note there may be regional differences in TDP43 pathology, with the FI VENs showing more TDP43 pathology compared to the VENs in the ACC.

### Clinical features

Our cohort shows no difference in the GABRQ‐expressing ratio within the C9‐bvFTD donors based on presence of memory, speech and MND symptoms in the first half of their disease course. Previously, a significant difference in number of VENs was found between donors with predominantly MND symptoms and bvFTD 10. Our cohort had three donors that reported MND symptoms in the first half of their disease duration, however, in all donors this was accompanied by behavioural symptoms; in this study no *C9orf72* donors were available with pure MND. Nonetheless, a lower ratio of GABRQ‐expressing neurons was observed in relation to the absence of MND symptoms compared to controls, indicating that the pure behavioural variant of C9‐bvFTD without MND is associated with a lower ratio of GABRQ‐expressing neurons. Concerning psychotic symptoms, we found similar results to Yang *et al*. 2017, where *C9orf72* donors with psychotic symptoms had a higher density of VENs compared to donors without psychotic symptoms 10. This observation also extends to the GABRQ‐expressing population, indicating that the GABRQ‐expressing population and VENs are not key‐regulators of psychotic symptoms in C9‐bvFTD.

### GABRQ can be specifically modulated

The findings of this study have impact on the therapeutic intervention of bvFTD. GABRQ is expressed in other regions of the brain, including hypothalamus, amygdala and brainstem structures such as periaqueductal grey and locus coeruleus 18, 34, 35, 36, 37. Possible subunit assembly partners of GABRQ have been studied in rat locus coeruleus where GABRQ is reported to be co‐expressed with GABRE 34. In the ACC and FI of the human cortex, a clear segregation of the GABA receptor subunits can be observed, with GABRE in layer 5a and GABRQ in layer 5b, indicating that the subunits are not co‐expressed in the human cortical population. This provides an opportunity for specific target modulation based on the unique GABA receptor subunit expression.

To conclude, we have shown that VENs are part of a larger neuronal population characterized by GABRQ expression. This population is selectively vulnerable in C9‐bvFTD and spared in typical amnestic AD neurodegeneration. This suggest that this neuronal population is a key modulator of social and emotional functioning. The selective vulnerability is found to be more prominent in donors with TDP43 type A laminar pathology, compared to type B and E, indicating that the distribution of TDP43 in the cortex determines the GABRQ‐expressing neuronal loss. These findings widen the possibilities of specific modulation of this neuronal population, as GABRQ appears to be expressed in a unique receptor subunit assembly combination.

## Author contributions

AAD and PGP designed the study and wrote the manuscript. PGP, IvD and AAD performed the experiments and quantification of the data. PGP and AAD analysed the data. JCvS, YALP and JJHM provided intellectual contribution and participated in discussion. AJMR and NBB were responsible for the autopsy material and pathological evaluation. All authors read and approved the final manuscript.

## Ethical approval

All procedures performed in the study were in accordance of the ethical standards of Amsterdam University Medical Centre location VUmc.

## Informed consent

All donors gave informed consent for the use of their tissue and medical files for research purposes.

## Supporting information


**Table S1.** Detailed demographic and clinical data of donors
**Figure S1.** GABRQ and GABRE expression in the ACC.
