# Supplementary material for: Von Economo neurons are part of a larger neuronal population that are selectively vulnerable in C9orf72 frontotemporal dementia
Source: Neuropathol Appl Neurobiol. 2019 Jun 10;45(7):671–80. doi: 10.1111/nan.12558 (PMC6915913; doi:10.1111/nan.12558)
Supplement: Supplementary file 1 — Table S1. Detailed demographic and clinical data of donors Figure S1. GABRQ and GABRE expression in the ACC. [file NAN-45-671-s001.docx]

**Supplementary Table 1.** Detailed demographic and clinical data of donors

|  | Diagnosis | Sex | Age of death | Disease Duration | Co-pathologies  (A, B, C score) |
| --- | --- | --- | --- | --- | --- |
| 1 | Control | F | 64 | n/a | 2,0,0 |
| 2 | Control | M | 55 | n/a | 2,0,0 |
| 3 | Control | F | 64 | n/a | 0,1,0 |
| 4 | Control | F | 60 | n/a | 0,0,0 |
| 5 | Control | M | 83 | n/a | 3,1,0 |
| 6 | Control | F | 60 | n/a | 0,0,0 |
| 7 | Control | F | 76 | n/a | 1,1,0 |
| 8 | Control | M | 68 | n/a | 0,0,0 |
| 9 | Control | F | 70 | n/a | 2,1,0 |
| 10 | Control | M | 86 | n/a | 0,1,0 |
| 11 | Control | M | 53 | n/a | 2,1,0 |
| 12 | Control | M | 51 | n/a | 1,0,0 |
| 13 | C9-bvFTD | F | 72 | 7 | 1,1,0 |
| 14 | C9-bvFTD | M | 75 | 6 | 0,1,0 |
| 15 | C9-bvFTD | F | 68 | 7 | 0,2,0 |
| 16 | C9-bvFTD | F | 40 | 5 | 0,1,0 |
| 17 | C9-bvFTD | F | 77 | 7 | 1,1,0 |
| 18 | C9-bvFTD | M | 60 | 6 | 0,1,0 |
| 19 | C9-bvFTD | F | 70 | 8 | 2,2,1 |
| 20 | C9-bvFTD | F | 67 | 6 | 2,2,2 |
| 21 | C9-bvFTD | M | 64 | 8 | 1,1,0 |
| 22 | C9-bvFTD | M | 75 | 7 | 2,2,1 |
| 23 | C9-bvFTD | F | 52 | 7 | 0,1,0 |
| 24 | C9-bvFTD | F | 64 | 6 | 2,2,0 |
| 25 | C9-bvFTD/MND | F | 75 | 7 | 0,1,0 |
| 26 | C9-bvFTD/MND | M | 65 | 2 | 1,1,0 |
| 27 | C9-bvFTD/MND | F | 66 | 11 | 2,2,1 |
| 28 | C9-bvFTD/MND | M | 59 | 7 | 0,1,0 |
| 29 | AD | M | 87 | 3 | 3,3,3 |
| 30 | AD | M | 96 | 7 | 3,3,3 |
| 31 | AD | F | 69 | 9 | 3,3,3 |
| 32 | AD | F | 89 | 7 | 3,3,3 |
| 33 | AD | M | 82 | 8 | 3,3,3 |
| 34 | AD | M | 73 | 10 | 3,3,3 |
| 35 | AD | F | 78 | 12 | 3,3,3 |
|  |  |  |  |  |  |


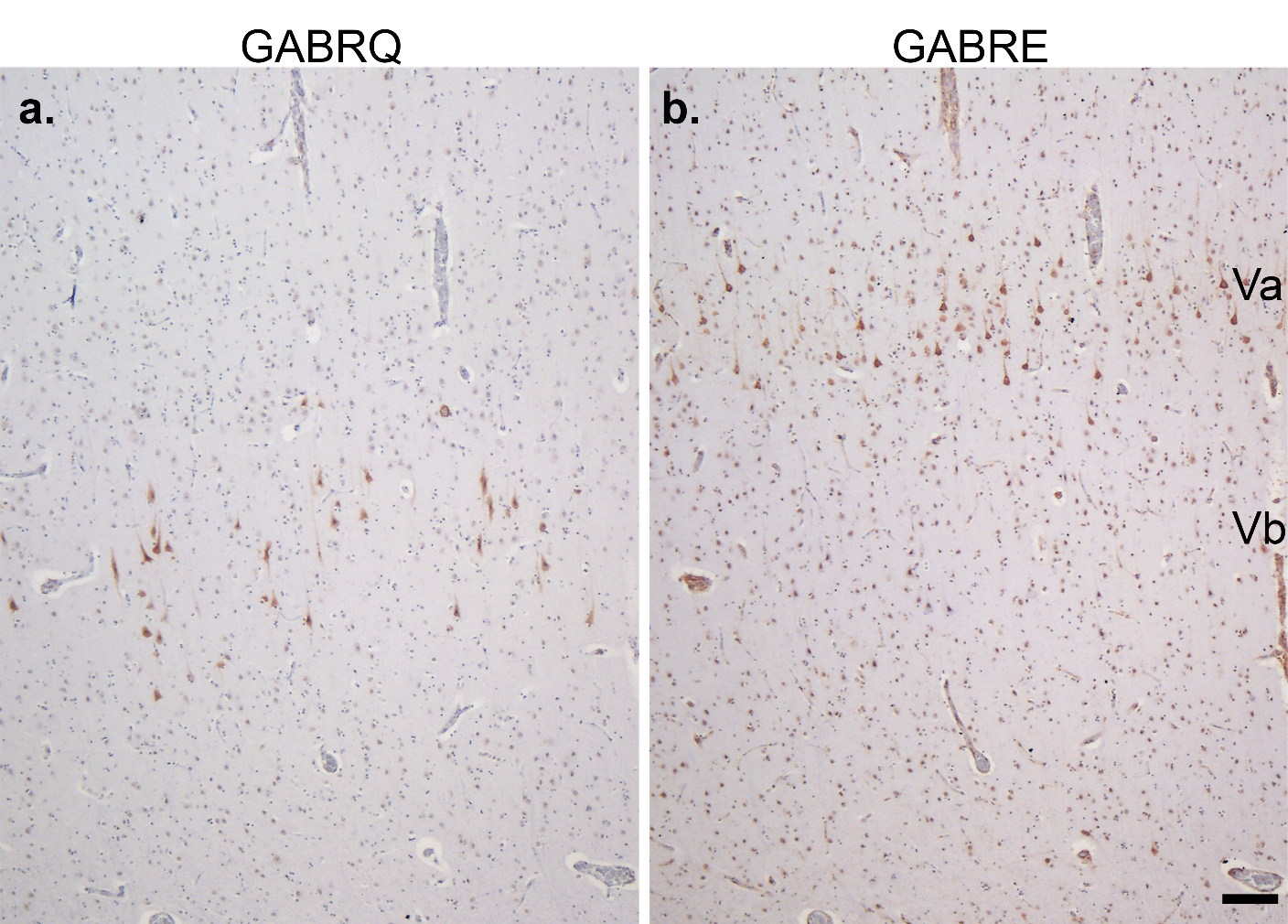


**Supplementary Fig. 1** GABRQ and GABRE expression in the ACC

GABRQ is expressed almost exclusively in layer 5b of the ACC (**a**), whereas GABRE is expressed in layer 5a (**b**). Sequential slides labelled for GABRQ and GABRE and were used to delineate layer 5 of the ACC, where GABRQ depicted the basal layer 5 border and GABRE the apical layer 5 border. Scale bar represent 50μm.
